# Crisis Communication About the Maui Wildfires on TikTok: Content Analysis of Engagement With Maui Wildfire–Related Posts Over 1 Year

**DOI:** 10.2196/67515

**Published:** 2025-03-04

**Authors:** Jim P Stimpson, Aditi Srivastava, Ketan Tamirisa, Joseph Keaweʻaimoku Kaholokula, Alexander N Ortega

**Affiliations:** 1 Department of Health Economics, Systems, and Policy, University of Texas Southwestern Medical Center, 5323 Harry Hines Blvd, Dallas, TX, 75390-9066, United States, 1 214-645-2567; 2 Peter O'Donnell Jr. School of Public Health, University of Texas Southwestern Medical Center, Dallas, TX, United States; 3 Frisco High School, Frisco, TX, United States; 4 Washington University in St. Louis, St Louis, MO, United States; 5 Department of Native Hawaiian Health, John A. Burns School of Medicine, University of Hawaiʻi at Mānoa, Honolulu, HI, United States; 6 Thompson School of Social Work & Public Health, University of Hawaiʻi at Mānoa, Honolulu, HI, United States

**Keywords:** social media, public health, disasters, Hawaii, media, post, communication, disaster, disaster communication, wildfire, information, dissemination, engagement, content analysis, content, metrics, misinformation, community, support

## Abstract

**Background:**

The August 2023 wildfire in the town of Lāhainā on the island of Maui in Hawaiʻi caused catastrophic damage, affecting thousands of residents, and killing 102 people. Social media platforms, particularly TikTok, have become essential tools for crisis communication during disasters, providing real-time crisis updates, mobilizing relief efforts, and addressing misinformation. Understanding how disaster-related content is disseminated and engaged with on these platforms can inform strategies for improving emergency communication and community resilience.

**Objective:**

Guided by Social-Mediated Crisis Communication theory, this study examined TikTok posts related to the Maui wildfires to assess content themes, public engagement, and the effectiveness of social media in disseminating disaster-related information.

**Methods:**

TikTok posts related to the Maui wildfires were collected from August 8, 2023, to August 9, 2024. Using TikTok’s search functionality, we identified and reviewed public posts that contained relevant hashtags. Posts were categorized into 3 periods: during the disaster (August 8 to August 31, 2023), the immediate aftermath (September 1 to December 31, 2023), and the long-term recovery (January 1 to August 9, 2024). Two researchers independently coded the posts into thematic categories, achieving an interrater reliability of 87%. Engagement metrics (likes and shares) were analyzed to assess public interaction with different themes. Multivariable linear regression models were used to examine the associations between log-transformed likes and shares and independent variables, including time intervals, video length, the inclusion of music or effects, content themes, and hashtags.

**Results:**

A total of 275 TikTok posts were included in the analysis. Most posts (132/275, 48%) occurred in the immediate aftermath, while 76 (27.6%) were posted during the long-term recovery phase, and 24.4% (n=67) were posted during the event. Posts during the event garnered the highest average number of likes (mean 75,092, SD 252,759) and shares (mean 10,928, SD 55,308). Posts focused on “Impact & Damage” accounted for the highest engagement, representing 36.8% (4,090,574/11,104,031) of total likes and 61.2% (724,848/1,184,049) of total shares. “Tourism Impact” (2,172,991/11,104,031, 19.6% of likes; 81,372/1,184,049, 6.9% of shares) and “Relief Efforts” (509,855/11,104,031, 4.6% of likes; 52,587/1,184,049, 4.4% of shares) were also prominent themes. Regression analyses revealed that videos with “Misinformation & Fake News” themes had the highest engagement per post, with a 4.55 coefficient for log-shares (95% CI 2.44-6.65), while videos about “Tourism Impact” and “Relief Efforts” also showed strong engagement (coefficients for log-likes: 2.55 and 1.76, respectively).

**Conclusions:**

TikTok is an influential tool for disaster communication, amplifying both critical disaster updates and misinformation, highlighting the need for strategic content moderation and evidence-based messaging to enhance the platform’s role in crisis response. Public health officials, emergency responders, and policy makers can leverage TikTok’s engagement patterns to optimize communication strategies, improve real-time risk messaging, and support long-term community resilience.

## Introduction

### Background

The wildfire that began on August 8, 2023, in the town of Lāhainā on Maui island in Hawaiʻi brought widespread destruction, disrupted the lives of residents, destroyed homes, killed 102 people, and altered the environment [1-4]. Wildfires are increasingly becoming a serious public health concern due to their devastating impacts on the environment, communities, and health infrastructure [5-7]. The growing frequency and intensity of wildfires have been, in part, attributed to climate change, which creates hotter and drier conditions conducive to fire outbreaks [8-10]. Understanding public perceptions and engagement with these disasters is crucial for enhancing response, recovery, and resiliency [11].

In the digital age, social media platforms have emerged as critical tools for crisis communication, particularly during natural disasters and public health emergencies, providing situational updates, relief information, emotional support, and avenues for public mobilization [12-19]. TikTok has emerged as a key platform for public engagement during crises, offering a unique short-form video format that encourages personal storytelling and emotional connection [20-26]. TikTok’s algorithm promotes high-engagement posts, which amplifies diverse perspectives. Previous studies, such as Sharma and De Choudhury’s [20] exploration of TikTok in public health, underscore its value in disseminating information and fostering community resilience. However, limited research has focused on its role during natural disasters, especially compared with platforms like Facebook or Instagram [13,14]. The rise of mis and disinformation on social media also poses challenges, which potentially complicates disaster response and relief efforts [27-31].

Despite TikTok’s increasing influence, few studies have focused on its role in communicating and shaping public discourse during natural disasters. One study analyzed TikTok videos from official accounts featured in the COVID-19 information hub, focusing on how credible information was disseminated to combat misinformation during the pandemic, highlighting the platform’s role in delivering accessible, authoritative public health messaging [32]. Another examined trending TikTok content during the early days of the US COVID-19 pandemic, emphasizing how the platform facilitated social connection and coping through creative and relatable content during the “shelter in place” period [33]. A third study evaluated TikTok videos tagged with #ClimateChange to assess the platform’s use in raising awareness and driving discussions on environmental issues [34]. Collectively, these studies underscore TikTok’s growing importance as a tool for disseminating information, fostering public engagement, and influencing perceptions on global challenges but more information is needed about its use during natural disasters.

This study applies Social-Mediated Crisis Communication (SMCC) theory to understand how TikTok was used to disseminate and engage with information about the August 2023 Maui wildfires. SMCC theory explains how people seek, share, and respond to crisis information in digital environments [35]. SMCC theory posits that during a crisis, individuals rely on traditional media, social media influencers, and peer networks to make sense of unfolding events. The theory also emphasizes the role of organizational crisis communicators, but in the case of decentralized platforms like TikTok, crisis-related information is largely driven by user-generated content rather than official sources. This shift highlights the need to analyze which themes and message characteristics drive engagement on social media during disasters [35,36].

Applying SMCC theory, this study examined how content themes, video features (eg, video length, music, and effects), and hashtags influence engagement metrics, including likes and shares. Specifically, we assessed whether certain types of crisis messages align with SMCC theory’s key information-sharing mechanisms. Instructing information refers to messages that provide practical guidance or factual updates, such as evacuation notices and safety alerts. Adjusting information includes messages that help users emotionally process the crisis, including personal narratives and community responses. Bolstering information consists of messages that reinforce community solidarity, fundraising efforts, and expressions of resilience. Prior research indicates that adjusting and bolstering messages often garner higher engagement on social media platforms, as users seek emotional support and opportunities to contribute to recovery efforts [36-38]. For instance, a study analyzing social media interactions during crises found that content providing emotional support and community solidarity received substantial user engagement [37]. Conversely, while instructing information—such as safety alerts and factual updates—is crucial for public safety, it tends to receive comparatively lower engagement, especially on entertainment-focused platforms like TikTok [23-26]. Research on TikTok’s user engagement patterns suggests that content aligning with entertainment and emotional resonance is more likely to engage users than purely informational posts [38].

By applying SMCC theory, this study seeks to identify which crisis communication strategies gain traction on TikTok, offering insights into how social media platforms shape public understanding and response during natural disasters. The findings have implications for emergency responders, public health officials, and community organizations aiming to optimize social media engagement in future crises. Natural disasters, like the Maui wildfire, are typically seen as external and uncontrollable, which places greater emphasis on the need for empathetic and supportive communication rather than blame attribution [35-38]. Understanding how social media users adapt these strategies in real time contributes to advancing best practices for disaster communication [12-15].

### Research Questions

Guided by SMCC theory, this study examined public engagement with TikTok content related to the Maui wildfire to understand its role in digital health communication during disasters. Specifically, we address three key questions: (1) how do TikTok posts related to the Maui wildfire align with SMCC’s instructing, adjusting, and bolstering information-sharing mechanisms and what are the implications for public health messaging during crises; (2) which content themes generate the highest engagement among TikTok users throughout different phases of the disaster and how do these themes contribute to public health awareness, misinformation spread, and community resilience; and (3) how does engagement evolve over time from the immediate disaster response to the long-term recovery period and what does this reveal about the role of social media for public health communication after the immediate danger of crisis is past? By examining these research questions, this study contributes to the growing body of literature on digital health communication in disaster contexts, focusing on TikTok as an influential platform shaping disaster-related communication strategies. Understanding public engagement patterns across distinct temporal phases provides actionable insights for public health agencies, emergency responders, and policy makers to strengthen community resilience, improve evidence-based health messaging, and optimize the use of social media platforms for disaster response and recovery.

## Methods

### Data Collection

We collected TikTok posts about the Maui wildfire from August 8, 2023, to August 9, 2024, covering both the immediate aftermath of the wildfire and the 1-year anniversary. This timeframe was chosen to align with the phases emphasized in SMCC theory, which highlights the evolving nature of crisis communication on social media, emphasizing both the acute crisis period and the extended postcrisis discourse that shapes public understanding of response and recovery [35]. Posts were identified using a combination of disaster-related hashtags, including #MauiStrong and #MauiWildfires, and were sampled until thematic saturation was achieved [39].

To ensure diverse and representative content, posts were selected at random from the top search results for each hashtag or hashtag combination, prioritizing those with high visibility. For each hashtag, all available posts were thoroughly reviewed and included if they were relevant to the wildfire. Duplicate posts were removed to prevent redundancy and posts unrelated to the wildfire were excluded. After reaching thematic saturation, the final data set consisted of 275 unique posts, which was sufficient to capture the breadth of content shared about the wildfire [39].

### Measures

The outcome variables for this study were likes and shares, which represent the primary engagement metrics for TikTok posts. Likes captured the number of times users expressed approval of a post, and shares indicated how often a post was redistributed by users to others on the platform. To address outliers in these engagement metrics, we applied a log transformation to both likes and shares. This transformation helped normalize the distribution of the variables and reduced the influence of extreme values, ensuring a more robust analysis. The log-transformed values were used as dependent variables in the regression models to evaluate factors influencing engagement.

Posts were coded with a variety of themes, and each post could be coded with multiple themes. Two researchers independently coded the posts to ensure reliability and minimize bias. Discrepancies in coding were resolved through discussion and consensus. The level of agreement between the 2 researchers was measured using the κ reliability statistic, which resulted in a high agreement rate of 87%. This high level of interrater reliability confirms the consistency and accuracy of the thematic classification [40].

We identified several major themes in the TikTok posts related to the Maui wildfire, each reflecting different aspects of the event and its aftermath. One prominent theme was “Impact & Damage”, with posts documenting the destruction caused by the wildfires. These posts often included powerful visuals, such as videos and images of affected areas, illustrating the extent of the devastation. The “Personal Narratives & Interviews” theme featured individuals sharing their experiences of living through the wildfire. These posts were often deeply emotional, with appeals for continued attention to the needs of those affected. Personal stories ranged from immediate reactions during the wildfire to reflections on the ongoing recovery process, amplifying the voices of those directly impacted by the disaster. The theme of “Community, Solidarity, & Tribute” was also widely represented. These posts conveyed messages of support and solidarity from people around the world. Many stories highlighted local heroes, community efforts, and the resilience of those affected by the wildfire. A common subtheme within these posts was the presentation of “before the fire” scenes, which offered a sentimental and solemn perspective on what was lost and underscored the emotional impact of the disaster.

Another key theme was “Relief Efforts”, which encompassed content aimed at promoting fundraisers, donations, and other forms of assistance. Posts in this category highlighted the work of both local and national organizations providing aid to affected communities. They also documented the progress (or lack thereof) in recovery efforts, which served as calls to action for further support from the public. These posts were integral in mobilizing resources and raising awareness about ongoing needs in the aftermath of the wildfire. Posts related to “Government & Policy Responses” critiqued or praised the governmental handling of the wildfire. Discussions centered on the effectiveness of emergency preparedness and response efforts, with users offering their perspectives on the adequacy of relief efforts, resource allocation, and communication from officials during and after the crisis. “Tourism Impact” was another important theme.

As Maui is a major tourist destination and most tourists to the island visit Lāhainā, many posts discussed how the wildfires affected the tourism industry. Both travelers and locals shared their thoughts on the changing landscape and economy, reflecting on how the wildfires disrupted the flow of visitors and altered the island’s economic prospects. Posts categorized under “Environmental & Climate Commentary” explored the role of climate change in exacerbating wildfires. Users discussed how changing weather patterns and prolonged droughts have contributed to the frequency and intensity of such natural disasters. Many of these posts also advocated for policy changes aimed at mitigating future risks, calling for stronger environmental protections and climate action to prevent similar events from occurring.

Finally, posts under the “Informational” theme provided detailed overviews of the wildfire. These posts explained the causes, effects, and other key details of the disaster, often serving as educational content for users looking to understand the broader context and implications of the wildfire. These posts helped disseminate factual and descriptive information about the event. In contrast, the theme of “Misinformation & Fake News” was also present. Posts categorized as “Misinformation” were identified based on 2 criteria: posts that discussed and attempted to debunk misinformation about the fires by explaining why the claims were inaccurate and posts that directly promoted misinformation, with the accuracy of their claims verified through fact-checking conducted by our research team. Any post meeting either of these criteria was classified as “Misinformation.”

We included several other measures to assess how structural and temporal characteristics of posts influenced engagement levels (Likes and Shares). Video length, representing the duration of each TikTok post, was measured in seconds and included in the analyses as a continuous variable. A squared term for video length was also included to account for potential nonlinearity in its relationship with engagement metrics. Additionally, the presence of music or effects was included as a binary variable, capturing whether a post used TikTok’s music or special effects features. Event timing was categorized into 3 periods: during the disaster (August 8 to August 31, 2023), the immediate aftermath (September 1 to December 31, 2023), and the long-term recovery (January 1 to August 9, 2024). Posts were coded based on their publication date relative to these time frames. This segmentation aligns with SMCC’s theoretical framework, enabling insights into how content types and engagement patterns shift over time [35].

We categorized individual hashtags into thematic groups to analyze engagement patterns related to the Maui wildfire. Each hashtag group was designed to capture distinct aspects of public discourse and response. Posts were assigned to groups based on predefined hashtags, with a binary variable indicating whether a post contained at least 1 relevant hashtag. The “Support” group included hashtags such as #MauiStrong, #PrayForMaui, and #MauiSupport, capturing expressions of solidarity and general support for those affected. The “Relief” group encompassed hashtags like #DonateForMaui, #Fundraiser, #MauiRelief, #DisasterRelief, and #RedCross, reflecting calls for donations, fundraising efforts, and relief activities. The “WildfireContent” group included hashtags such as #MauiWildfires, #Wildfire, #WildfireAwareness, and #FirePrevention, focusing on wildfire-specific content, including awareness and prevention efforts. The “SafetyAlerts” group comprised hashtags like #StaySafe and #EmergencyAlert, emphasizing safety information and emergency updates. The “MauiLocations” group included hashtags referencing specific locations such as #Lahaina, #Kaanapali, #WestMaui, and #Hawaii, highlighting geographically specific content. The “AdvocacyAndUpdates” group encompassed hashtags such as #EnvironmentalJustice, #NewsUpdate, and #BreakingNews, reflecting advocacy-related posts and updates on unfolding events. Each group was assigned a binary variable, where a post was flagged as belonging to a group if it contained at least one of the associated hashtags. Logical functions in spreadsheet software were used to implement this classification efficiently, which ensured consistent assignment of posts to their respective groups.

### Data Analysis

Engagement metrics were analyzed to assess immediate reactions and the reach of content to viewers [41,42]. The analysis strategy began with descriptive statistics to provide an overview of the distribution of engagement metrics (likes and shares) across key categories, such as time intervals and hashtag groups. We summarized engagement metrics by time interval, showing the number of posts, average likes and shares, and their distribution across the event duration, immediate aftermath, and long-term recovery phases. Crosstabulations of hashtags and content themes were conducted to examine how engagement metrics varied across different combinations of hashtag groups and content themes. These tables found in Multimedia Appendix 1 highlighted the total and relative contributions of each category to overall engagement.

The multivariable analyses included linear regression models predicting the log-transformed likes and shares. Independent variables included time intervals, video length (and its squared term), the presence of music or effects, content themes, and hashtag groups. This approach assessed the relative contribution of these variables to engagement metrics while adjusting for potential confounding factors. To test the robustness of the results, we replicated the linear regression models using robust regression, which is less sensitive to outliers and provides an alternative estimation strategy. The results of the robustness check in Multimedia Appendix 2 were largely consistent with the primary analyses, confirming the stability of key findings. This study adheres to the STROBE (Strengthening the Reporting of Observational studies in Epidemiology) guidelines for reporting observational studies.

### Ethical Considerations

The University of Hawaiʻi’s Office of Research Compliance determined that the study does not meet the definition of human participants research and therefore does not require institutional review board approval or oversight.

## Results

The analysis shown in Table 1 examined social media engagement metrics across the event time intervals. A total of 275 observations were included in the analysis. Most posts (132/275, 48%) occurred in the immediate aftermath, while 76 (27.6%) were posted during the long-term recovery phase, and 24.4% (n=67) were posted during the event. Posts during the event garnered the highest average number of likes, with a mean of 75,092 (SD 252,759). Engagement declined in subsequent periods, with posts in the “Immediate Aftermath” receiving an average of 40,641 likes, and posts in the “Long-Term Recovery” averaging 8685 likes. Across all intervals, the average number of likes was 40,203. Similarly, posts during the event achieved the highest average number of shares, with a mean of 10,928 (SD 55,308). This figure dropped to 3111 in the “Immediate Aftermath” and further declined to 493 in the “Long-Term Recovery” period. The overall average number of shares across all intervals was 4292.

**Table 1.** Engagement metrics (likes and shares) by time interval. Content analysis of 275 TikTok posts from August 8, 2023, to August 9, 2024.

	Value, n (%)	Likes, mean (SD)	Shares, mean (SD)
During event (August 8 to August 31, 2023)	67 (24.4)	75,092 (252,759)	10,928 (55,308)
Immediate aftermath (September 1 to December 31, 2023)	132 (48.0)	40,641 (184,994)	3111 (10,365)
Long-term recovery (January 1 to August 9, 2024)	76 (27.6)	8685 (27,244)	493 (1,207)
Total	275 (100.0)	124,418	14,532

Table 2 presents the results from the linear regression analysis of log-transformed likes. Video length was positively associated with likes, with the linear term (coefficient=0.01, *P*=.05) indicating a slight increase in engagement for longer videos. However, the quadratic term (coefficient=0.00, *P*=.03) suggests diminishing returns for extremely long videos. Event timing showed that engagement was significantly lower during the immediate aftermath of the disaster (coefficient=−0.96, *P*=.01) and during the long-term recovery phase (coefficient=−1.47, *P*<.001) compared with the disaster period. Videos featuring music or effects had a marginally lower engagement, though this relationship was not statistically significant (coefficient=−0.56, *P*=.09). Regarding content themes, several categories significantly increased engagement. Videos focused on “Impact & Damage” (coefficient=1.55, *P*=.01), “Informational” content (coefficient=1.32, *P*=.04), “Misinformation & Fake News” (coefficient=3.24, *P*<.001), “Personal Narratives & Interviews” (coefficient=1.66, *P*=.02), “Relief Efforts” (coefficient=1.62, *P*=.02), and “Tourism Impact” (coefficient=2.53, *P*<.001) all received higher levels of likes compared with the reference group. In contrast, themes such as “Environmental & Climate Commentary” and “Government & Policy Response” did not significantly impact engagement. For hashtags, the “Support” and “WildfireContent” groups were associated with significantly higher engagement, with coefficients of 1.90 (*P*<.001) and 1.87 (*P*<.001), respectively, compared with the reference group. However, the “MauiLocations” and “Relief” hashtags did not exhibit significant effects.

**Table 2.** Multivariable linear regression of log-transformed likes. Content analysis of 275 TikTok posts from August 8, 2023, to August 9, 2024.

		Coefficient	*P* value	95% CI
Video length		0.01	.05	0.00 to 0.02
Video length square		0.00	.03	0.00 to 0.00
Event timing				
	During disaster	—^a^	—	—
	Immediate aftermath	−0.96	.01	−1.71 to −0.21
	Long-term recovery	−1.47	<.001	−2.31 to −0.63
Music and effects		−0.56	.09	−1.21 to 0.09
Content theme				
	Community, Solidarity, & Tribute	—	—	—
	Environmental & Climate Commentary	0.04	.97	−2.19 to 2.27
	Government & Policy Response	0.95	.23	−0.61 to 2.51
	Impact & Damage	1.55	.01	0.33 to 2.77
	Informational	1.32	.04	0.05 to 2.58
	Misinformation & Fake News	3.24	<.001	1.22 to 5.26
	Personal Narratives & Interviews	1.66	.02	0.32 to 3.00
	Relief Efforts	1.62	.02	0.23 to 3.00
	Tourism Impact	2.53	<.001	1.02 to 4.05
Hashtag group				
	AdvocacyAndUpdates	—	—	—
	MauiLocations	0.00	>0.99	−0.89 to 0.90
	Relief	−0.93	.12	−2.09 0.24
	Support	1.90	<.001	0.97 to 2.84
	WildfireContent	1.87	<.001	0.95 to 2.78
Constant		6.02	<.001	4.55 to 7.49

a Not applicable (reference category).

Table 3 presents the linear regression analysis for log-transformed shares. Video length was not significantly associated with shares. The Immediate Aftermath period was negatively associated with shares (coefficient=−0.82, *P*=.04), and long-term recovery showed an even stronger negative effect (coefficient=−1.51, *P*<.001), both indicating fewer shares during these periods compared with the baseline (during event). Content themes also had significant effects on shares. Specifically, “Impact & Damage,” “Informational,” and “Misinformation & Fake News” content themes had large positive associations with shares (coefficients of 2.46, 1.86, and 4.55, respectively, all with *P* values <.01), suggesting that content related to these themes is shared more frequently. “Personal Narratives & Interviews,” “Relief Efforts,” and “Tourism Impact” also showed significant positive associations with shares, further emphasizing the popularity of content related to these themes. Finally, certain hashtag groups influenced shares. “Relief” had a negative impact on shares (coefficient=−1.68, *P*=.01), while “Support” and “WildfireContent” both had positive effects (coefficients of 2.28 and 1.64, respectively, both with *P* values <.01), indicating that these types of hashtags contribute to higher share rates relative to the reference group.

**Table 3.** Multivariable linear regression of log-transformed shares. Content analysis of 275 TikTok posts from August 8, 2023, to August 9, 2024.

		Coefficient	*P* value	95% CI
Video length		0.01	.08	0.00 to 0.02
Video length square		0.00	.08	0.00 to 0.00
Event timing				
	During disaster	—^a^	—	—
	Immediate aftermath	−0.82	.04	−1.60 to −0.04
	Long-term recovery	−1.51	<.001	−2.39 to −0.64
Music and effects		−0.43	.21	−1.11 to 0.25
Content theme				
	Community, Solidarity, & Tribute	—	—	—
	Environmental & Climate Commentary	0.44	.71	−1.89 to 2.77
	Government & Policy Response	1.62	.05	0.00 to 3.25
	Impact & Damage	2.46	<.001	1.19 to 3.73
	Informational	1.86	.01	0.54 to 3.18
	Misinformation & Fake News	4.55	<.001	2.44 to 6.65
	Personal Narratives & Interviews	2.11	<.001	0.72 to 3.51
	Relief Efforts	2.38	<.001	0.94 to 3.82
	Tourism Impact	2.96	<.001	1.37 to 4.54
Hashtag group				
	AdvocacyAndUpdates	—	—	—
	MauiLocations	0.19	.70	−0.75 to 1.12
	Relief	−1.68	.01	−2.89 to −0.46
	Support	2.28	<.001	1.30 to 3.25
	WildfireContent	1.64	<.001	0.68 to 2.60
Constant		2.70	<.001	1.17 to 4.23

a Not applicable (reference category).

## Discussion

### Principal Findings

This study provides novel insights into how TikTok serves as a platform for public engagement during and after a natural disaster, using the 2023 Maui wildfire as a case study. Guided by SMCC theory, we examined how content themes, hashtags, and video features influence user engagement metrics, such as likes and shares. Our findings contribute to the growing body of literature on social media’s role in crisis communication while offering unique contributions that expand upon prior studies [43-49].

This study reveals that TikTok serves as a dynamic platform for real-time information dissemination and public engagement during and after natural disasters. Posts emphasizing themes such as “Impact & Damage” and “Personal Narratives & Interviews” garnered the highest engagement metrics, illustrating the platform’s capacity to emotionally and visually document the crisis. Content highlighting tourism and economic impacts also performed strongly, reflecting the broader public’s interest in understanding the disaster’s ripple effects. However, posts addressing critical but less emotional themes, such as “Government & Policy Response” and “Relief Efforts,” attracted relatively lower engagement, highlighting a potential gap in how these topics resonate with TikTok audiences.

Engagement trends varied across the disaster timeline. While a prior study on the Maui wildfire focused on the immediate aftermath of the disaster, we analyzed engagement over a 1-year period, encompassing both crisis and postcrisis phases [38]. This extended time frame enabled us to explore the evolution of public engagement and highlighted key shifts in user priorities over time. Posts shared during the crisis phase generated the highest engagement, underscoring the public’s urgent demand for real-time updates. Engagement declined in the immediate aftermath and long-term recovery periods, reflecting waning public attention. These patterns emphasize the importance of strategically timing communication to sustain public interest and engagement throughout the recovery phase. The observed temporal trends also underscore the need for innovative strategies to re-engage audiences as the crisis progresses into recovery [35].

Engagement was predominantly driven by themes that conveyed the emotional impact and visual magnitude of the disaster, reinforcing the role of emotionally resonant content in shaping public discourse [37,38]. This pattern aligns with SMCC theory, which emphasizes the importance of adjusting information in crisis communication—content that helps audiences process the event and fosters emotional connection [35]. Moreover, informational posts about safety alerts and evacuation notices garnered relatively low engagement despite their critical importance for public safety. In contrast, adjusting and bolstering information, such as personal narratives and fundraising efforts, received significantly higher engagement, reflecting the public’s preference for emotionally resonant and actionable content. These findings suggest that while instructing information is vital during the early crisis phase, sustained engagement may rely on content that fosters emotional connection and community solidarity [35,36].

Our results underscore the importance of visually compelling and emotionally resonant content in driving engagement during disasters [50-52]. This aligns with a recent study that found audiovisual vividness was a key predictor of engagement on TikTok during the same wildfire [38]. Both studies highlight the role of survivor-led narratives in capturing public attention, suggesting that user-generated content may resonate more strongly than traditional news media posts. However, while prior work used a digital storytelling framework to analyze the role of narrative persuasion and visual storytelling, our study extends the literature by applying SMCC theory to examine how instructing, adjusting, and bolstering messages align with engagement patterns [35-38]. This distinction situates our findings within the broader literature on crisis communication strategies.

Furthermore, our study highlights the persistent challenge of misinformation during disasters [21,27-29,53]. Posts categorized under misinformation themes, while limited in number, garnered substantial engagement, a finding that aligns with a recent study that found that posts leveraging emotional storytelling and high audiovisual vividness were particularly effective at drawing audience engagement [38]. This finding underscores the broader concern in public health regarding the virality of misinformation on social media, where false claims about disaster response, relief efforts, or health risks can undermine public trust in official sources [27-31]. Proactive strategies are needed to combat misinformation and amplify credible sources during crisis events. Integrating insights from both SMCC theory and the digital storytelling literature, our findings suggest that pairing information with compelling visual narratives may enhance its reach and impact, particularly on entertainment-driven platforms like TikTok.

Finally, our study contributes to the ongoing discourse on the role of video features in driving engagement [25,26,50,51]. The platform’s algorithm-driven reach and short-form video format provide unique opportunities to disseminate impactful messages that resonate with diverse audiences [34,38,54]. Consistent with prior research, we find that shorter videos with clear and emotionally engaging messages are more likely to capture public attention [55]. These findings align with broader trends in TikTok engagement metrics and highlight the need for tailored communication strategies that align with platform-specific user behaviors. Tailored strategies to amplify these themes could enhance the platform’s utility in disaster response [20].

### Limitations

While 2 researchers independently coded the posts to ensure reliability and minimize bias, the interpretation of themes still involves subjective judgment. Even with our high interrater reliability, some residual bias may persist. The 1-year timeframe of the study provides a snapshot but might miss evolving trends over a longer duration. Additionally, the focus on TikTok, with its distinct user demographics and content styles, limits the generalizability of the findings to other social media platforms such as X (formerly Twitter), Facebook, or Instagram. The unique characteristics of TikTok’s user base might not reflect broader social media trends. Furthermore, language and cultural barriers could have resulted in the underrepresentation of posts in languages other than English or those deeply rooted in specific cultural contexts, limiting the diversity of perspectives analyzed.

We acknowledge that TikTok’s algorithmic influence on post visibility is a significant factor, as the platform curates content based on user behavior, preferences, and engagement, potentially creating biases in the posts available for analysis. As a result, our data set may disproportionately reflect content with high algorithmic visibility rather than a fully representative sample of all relevant posts. Additionally, regional and language biases in content discovery may affect the results, as TikTok’s algorithms and search functionalities prioritize content tailored to specific geographic regions and linguistic preferences. This could result in the underrepresentation of videos from certain regions or in languages less prominent on the platform. The impact of paid promotion or boosted content is another limitation. Videos that received paid promotions may have artificially higher visibility and engagement, skewing the data toward commercially sponsored content. Our study did not distinguish between organically popular posts and those boosted through paid promotion, which could influence the analysis of engagement trends.

### Implications for Future Research and Practice

The findings have important implications for disaster communication and public health response strategies. Understanding how engagement evolves over time and informs messaging that sustains public interest beyond the acute crisis phase. Disaster communicators can leverage TikTok’s ability to amplify emotionally resonant content while addressing misinformation through targeted campaigns. Future research should explore cross-platform comparisons to identify complementary roles of different social media platforms. Additionally, research on the effectiveness of public health interventions in countering misinformation and improving digital health literacy is needed to develop best practices for social media–based disaster communication.

### Conclusion

In conclusion, this study builds upon prior work by integrating theoretical insights from SMCC theory with empirical analyses of TikTok engagement during a major natural disaster. TikTok is an influential tool for real-time communication, emotional support, and advocacy during natural disasters. By examining themes related to the Maui wildfire, this study demonstrated how users respond to and interact with crisis-related content in real time. While TikTok’s strengths lie in its ability to visually and emotionally connect with audiences, addressing challenges such as misinformation and sustaining engagement throughout the recovery phase is essential. Strengthening digital crisis communication strategies, particularly in sustaining engagement beyond the immediate response phase, is critical for improving disaster preparedness and recovery efforts. Future research should further investigate best practices for leveraging social media in disaster preparedness, response, and community resilience.

## Supplementary material

10.2196/67515 Multimedia Appendix 1 Distribution of likes and shares.

10.2196/67515 Multimedia Appendix 2 Multivariable robust regression of log-transformed likes and shares.

## Abbreviations

SMCC: Social-Mediated Crisis Communication
STROBE: Strengthening the Reporting of Observational studies in Epidemiology
