# Supplementary material for: Crisis Communication About the Maui Wildfires on TikTok: Content Analysis of Engagement With Maui Wildfire–Related Posts Over 1 Year
Source: JMIR Form Res. 2025 Mar 4;9:e67515. doi: 10.2196/67515 (PMC11895724; doi:10.2196/67515)
Supplement: Multimedia Appendix 1 [file formative-v9-e67515-s001.docx]

Appendix 1

Table S1: Distribution of Likes by Content Theme and Hashtag Group (% of Total Likes): Content Analysis of 275 TikTok Posts, August 8 2023 – August 9 2024

|  | Hashtag Group | | | | |  |
| --- | --- | --- | --- | --- | --- | --- |
|  | AdvocacyAndUpdates | MauiLocations | Relief | Support | WildfireContent | Total |
| Content Theme |  |  |  |  |  |  |
| Community Solidarity & Tribute |  |  |  |  |  |  |
| N | 3,337 | 749 |  | 126,886 | 116,212 | 247,184 |
| % | 0.0 | 0.0 |  | 1.1 | 1.1 | 2.2 |
| Environmental & Climate Commentary |  |  |  |  |  |  |
| N | 12,451 | 80 |  | 5,807 |  | 18,338 |
| % | 0.1 | 0.0 |  | 0.1 |  | 0.2 |
| Government & Policy Response |  |  |  |  |  |  |
| N | 2,356 | 231,675 | 690 |  | 162,706 | 397,427 |
| % | 0.0 | 2.1 | 0.0 |  | 1.5 | 3.6 |
| Impact & Damage |  |  |  |  |  |  |
| N | 351,142 | 595,331 | 375 | 3,013,560 | 130,166 | 4,090,574 |
| % | 3.2 | 5.4 | 0.0 | 27.1 | 1.2 | 36.8 |
| Informational |  |  |  |  |  |  |
| N | 70,615 | 188,068 | 130 | 352,671 | 800,318 | 1,411,802 |
| % | 0.6 | 1.7 | 0.0 | 3.2 | 7.2 | 12.7 |
| Misinformation & Fake News |  |  |  |  |  |  |
| N | 25,600 | 59,776 |  | 631 |  | 86,007 |
| % | 0.2 | 0.5 |  | 0.0 |  | 0.8 |
| Personal Narratives & Interviews |  |  |  |  |  |  |
| N | 7,851 | 232,767 | 90,650 | 512,558 | 1,326,027 | 2,169,853 |
| % | 0.1 | 2.1 | 0.8 | 4.6 | 11.9 | 19.5 |
| Relief Efforts |  |  |  |  |  |  |
| N | 55,020 | 74,947 | 140,464 | 157,406 | 82,018 | 509,855 |
| % | 0.5 | 0.7 | 1.3 | 1.4 | 0.7 | 4.6 |
| Tourism Impact |  |  |  |  |  |  |
| N | 17,206 | 162,291 | 61,713 | 1,905,377 | 26,404 | 2,172,991 |
| % | 0.2 | 1.5 | 0.6 | 17.2 | 0.2 | 19.6 |
| Total |  |  |  |  |  |  |
| N | 545,578 | 1,545,684 | 294,022 | 6,074,896 | 2,643,851 | 11,104,031 |
| % | 4.9 | 13.9 | 2.7 | 54.7 | 23.8 | 100.0 |

The bivariable analysis in Table S1 highlights notable overlaps between hashtag groups and content themes in relation to likes. The "Support" hashtag group, which accounted for the majority of likes (54.7%), showed substantial engagement with the "Impact & Damage" theme, contributing 27.1% of total likes. Similarly, the "Tourism Impact" theme, which accounted for 19.6% of overall likes, was predominantly driven by the "Support" hashtag group (17.2%). The "WildfireContent" hashtag group, representing 23.8% of likes, exhibited strong engagement with "Informational" content (7.2%) and "Personal Narratives & Interviews" (11.9%). In contrast, "MauiLocations," accounting for 13.9% of total likes, was notably associated with "Impact & Damage" (5.4%). Themes such as "Government & Policy Response" and "Relief Efforts" were more aligned with specific hashtag groups, like "MauiLocations" and "Relief," but had relatively lower engagement overall. Minimal engagement was observed for "Community Solidarity & Tribute" and "Environmental & Climate Commentary," which collectively contributed less than 2.5% of total likes. This analysis underscores the differential impact of hashtag groups across content themes, revealing distinct patterns in public engagement during the Maui wildfires.

Table S2: Distribution of Shares by Content Theme and Hashtag Group (% of Total Shares): Content Analysis of 275 TikTok Posts, August 8 2023 – August 9 2024

|  | Hashtag Group | | | | |  |
| --- | --- | --- | --- | --- | --- | --- |
|  | AdvocacyAndUpdates | MauiLocations | Relief | Support | WildfireContent | Total |
| Content Theme |  |  |  |  |  |  |
| Community Solidarity & Tribute |  |  |  |  |  |  |
| N | 51 | 69 |  | 5,472 | 1,159 | 6,751 |
| % | 0.0 | 0.0 |  | 0.5 | 0.1 | 0.6 |
| Environmental & Climate Commentary |  |  |  |  |  |  |
| N | 1,447 | 1 |  | 599 |  | 2,047 |
| % | 0.1 | 0.0 |  | 0.1 |  | 0.2 |
| Government & Policy Response |  |  |  |  |  |  |
| N | 221 | 30,825 | 29 |  | 10,292 | 41,367 |
| % | 0.0 | 2.6 | 0.0 |  | 0.9 | 3.5 |
| Impact & Damage |  |  |  |  |  |  |
| N | 57,123 | 16,706 | 8 | 630,515 | 20,496 | 724,848 |
| % | 4.8 | 1.4 | 0.0 | 53.3 | 1.7 | 61.2 |
| Informational |  |  |  |  |  |  |
| N | 2,018 | 13,454 | 22 | 28,679 | 49,416 | 93,589 |
| % | 0.2 | 1.1 | 0.0 | 2.4 | 4.2 | 7.9 |
| Misinformation & Fake News |  |  |  |  |  |  |
| N | 1,608 | 7,301 |  | 309 |  | 9,218 |
| % | 0.1 | 0.6 |  | 0.0 |  | 0.8 |
| Personal Narratives & Interviews |  |  |  |  |  |  |
| N | 853 | 3,349 | 4,204 | 47,375 | 116,489 | 172,270 |
| % | 0.1 | 0.3 | 0.4 | 4.0 | 9.8 | 14.6 |
| Relief Efforts |  |  |  |  |  |  |
| N | 6,273 | 6,440 | 25,510 | 9,311 | 5,053 | 52,587 |
| % | 0.5 | 0.5 | 2.2 | 0.8 | 0.4 | 4.4 |
| Tourism Impact |  |  |  |  |  |  |
| N | 659 | 16,524 | 1,724 | 61,984 | 481 | 81,372 |
| % | 0.1 | 1.4 | 0.2 | 5.2 | 0.0 | 6.9 |
| Total |  |  |  |  |  |  |
| N | 70,253 | 94,669 | 31,497 | 784,244 | 203,386 | 1,184,049 |
| % | 5.9 | 8.0 | 2.7 | 66.2 | 17.2 | 100.0 |

Table S2 presents the cross-tabulation of Shares across hashtag groups and content themes, highlighting key overlaps. The "Support" hashtag group accounted for 66.2% of all Shares. This was driven primarily by "Impact & Damage," which contributed 53.3% of the total Shares within this group. The "WildfireContent" hashtag group, which made up 17.2% of total Shares, was also largely driven by "Impact & Damage," though "Personal Narratives & Interviews" and "Informational" content contributed notably within this group at 9.8% and 4.2%, respectively. The "MauiLocations" group, representing 8.0% of Shares overall, saw substantial engagement with "Government & Policy Response" (2.6%) and "Impact & Damage" (1.4%). Meanwhile, "AdvocacyAndUpdates," accounting for 5.9% of total Shares, was primarily associated with "Impact & Damage" (4.8%). Within the content themes, "Impact & Damage" emerged as the most significant driver of Shares across multiple hashtag groups, contributing 61.2% of total Shares. Other notable themes included "Personal Narratives & Interviews," which garnered 14.6% of Shares, with the majority coming from the "WildfireContent" and "Support" groups, and "Informational" content, which accounted for 7.9% of Shares, with strong contributions from the "WildfireContent" and "Support" groups. Smaller but notable contributions came from "Relief Efforts" (4.4%) and "Tourism Impact" (6.9%), with the latter being particularly prominent within the "Support" group (5.2%). This analysis underscores the concentration of Shares in content related to disaster impact, personal stories, and informational updates within specific hashtag groups.
