# Supplementary material for: Crisis Communication About the Maui Wildfires on TikTok: Content Analysis of Engagement With Maui Wildfire–Related Posts Over 1 Year
Source: JMIR Form Res. 2025 Mar 4;9:e67515. doi: 10.2196/67515 (PMC11895724; doi:10.2196/67515)
Supplement: Multimedia Appendix 2 [file formative-v9-e67515-s002.docx]

Appendix 2

Table S3: Multivariable Robust Regression of Log-Transformed Likes and Shares: Content Analysis of 275 TikTok Posts, August 8 2023 – August 9 2024

|  | Log Likes | | | | Log Shares | | | |
| --- | --- | --- | --- | --- | --- | --- | --- | --- |
|  | Coefficient | P-value | Lower 95% CI | Upper 95% CI | Coefficient | P-value | Lower 95% CI | Upper 95% CI |
| Video Length | 0.01 | 0.04 | 0.00 | 0.02 | 0.01 | 0.07 | 0.00 | 0.02 |
| Video Length Square | 0.00 | 0.02 | 0.00 | 0.00 | 0.00 | 0.07 | 0.00 | 0.00 |
| Event Timing |  |  |  |  |  |  |  |  |
| During Disaster | Reference |  |  |  | Reference |  |  |  |
| Immediate Aftermath | -1.04 | 0.01 | -1.83 | -0.24 | -0.97 | 0.02 | -1.79 | -0.14 |
| Long-term Recovery | -1.46 | 0.00 | -2.35 | -0.57 | -1.53 | 0.00 | -2.45 | -0.61 |
| Music and/or Effects | -0.58 | 0.10 | -1.27 | 0.11 | -0.44 | 0.23 | -1.16 | 0.28 |
| Content Theme |  |  |  |  |  |  |  |  |
| Community Solidarity & Tribute | Reference |  |  |  | Reference |  |  |  |
| Environmental & Climate Commentary | 0.12 | 0.92 | -2.24 | 2.49 | 0.65 | 0.60 | -1.81 | 3.10 |
| Government & Policy Response | 1.06 | 0.21 | -0.60 | 2.71 | 1.70 | 0.05 | -0.01 | 3.42 |
| Impact & Damage | 1.64 | 0.01 | 0.35 | 2.94 | 2.54 | 0.00 | 1.20 | 3.88 |
| Informational | 1.48 | 0.03 | 0.14 | 2.83 | 2.10 | 0.00 | 0.70 | 3.49 |
| Misinformation & Fake News | 3.49 | 0.00 | 1.35 | 5.63 | 4.68 | 0.00 | 2.46 | 6.90 |
| Personal Narratives & Interviews | 1.77 | 0.02 | 0.35 | 3.19 | 2.21 | 0.00 | 0.74 | 3.68 |
| Relief Efforts | 1.76 | 0.02 | 0.29 | 3.22 | 2.50 | 0.00 | 0.98 | 4.03 |
| Tourism Impact | 2.55 | 0.00 | 0.94 | 4.16 | 2.98 | 0.00 | 1.31 | 4.65 |
| Hashtag Group |  |  |  |  |  |  |  |  |
| AdvocacyAndUpdates | Reference |  |  |  | Reference |  |  |  |
| MauiLocations | -0.10 | 0.83 | -1.05 | 0.84 | 0.16 | 0.75 | -0.82 | 1.14 |
| Relief | -1.05 | 0.09 | -2.29 | 0.18 | -1.87 | 0.00 | -3.15 | -0.59 |
| Support | 1.99 | 0.00 | 1.00 | 2.98 | 2.37 | 0.00 | 1.34 | 3.40 |
| WildfireContent | 1.93 | 0.00 | 0.95 | 2.90 | 1.74 | 0.00 | 0.73 | 2.75 |
| Constant | 5.89 | 0.00 | 4.33 | 7.45 | 2.63 | 0.00 | 1.02 | 4.25 |

The robustness check analysis using robust regression is shown in Table S3 and largely confirms the results from the original linear regression models for log Likes and log Shares. For log Likes, the robust regression reaffirms that video length and its squared term have positive but modest associations with Likes, with p-values of 0.04 and 0.02, respectively. The Immediate Aftermath and Long-term Recovery periods are significantly associated with fewer Likes, with coefficients of -1.04 and -1.46, respectively, both achieving statistical significance (p < 0.05). Several content themes continue to significantly increase Likes, including Impact & Damage (coefficient = 1.64, p = 0.01), Informational (coefficient = 1.48, p = 0.03), Misinformation & Fake News (coefficient = 3.49, p < 0.01), Personal Narratives & Interviews (coefficient = 1.77, p = 0.02), Relief Efforts (coefficient = 1.76, p = 0.02), and Tourism Impact (coefficient = 2.55, p < 0.01). Additionally, hashtag groups such as Support (coefficient = 1.99, p < 0.01) and WildfireContent (coefficient = 1.93, p < 0.01) remain positively associated with Likes. For log shares, the robust regression analysis highlights a similar pattern of positive associations with video length (p = 0.07) and its squared term (p = 0.07). The Immediate Aftermath and Long-term Recovery periods continue to be negatively associated with Shares, with coefficients of -0.97 and -1.53, respectively, both achieving statistical significance (p < 0.05). Significant increases in Shares are associated with several content themes, including Impact & Damage (coefficient = 2.54, p < 0.01), Informational (coefficient = 2.10, p < 0.01), Misinformation & Fake News (coefficient = 4.68, p < 0.01), Personal Narratives & Interviews (coefficient = 2.21, p < 0.01), Relief Efforts (coefficient = 2.50, p < 0.01), and Tourism Impact (coefficient = 2.98, p < 0.01). Among hashtag groups, Support (coefficient = 2.37, p < 0.01) and WildfireContent (coefficient = 1.74, p < 0.01) show significant positive associations with Shares, while Relief is negatively associated (coefficient = -1.87, p < 0.01). The results provide additional confidence in the robustness of the findings, highlighting that themes such as Misinformation & Fake News, Impact & Damage, and Tourism Impact, as well as hashtags such as Support and WildfireContent, consistently drive engagement across both Likes and Shares. Negative associations with the Immediate Aftermath and Long-term Recovery periods are also consistently observed.
